# White Magnetic Paper with Zero Remanence Based on Electrospun Cellulose Microfibers Doped with Iron Oxide Nanoparticles

**DOI:** 10.3390/nano10030517

**Published:** 2020-03-12

**Authors:** G. Papaparaskeva, M. M. Dinev, T. Krasia-Christoforou, R. Turcu, S. A. Porav, F. Balanean, V. Socoliuc

**Affiliations:** 1 Department of Mechanical and Manufacturing Engineering, University of Cyprus, P.O. Box 20537, 1678 Nicosia, Cyprus; papaparaskeva.georgia@ucy.ac.cy (G.P.); marinov-dinev.marian@ucy.ac.cy (M.M.D.); krasia@ucy.ac.cy (T.K.-C.); 2 National Institute for Research and Development of Isotopic and Molecular Technologies, Donat 67-103, 400293 Cluj-Napoca, Romania; rodica.turcu@itim-cj.ro (R.T.); poravsebastian@gmail.com (S.A.P.); 3 Laboratory of Magnetic Fluids, Center for Fundamental and Advanced Technical Research, Romanian Academy—Timisoara Branch, M. Viteazul Ave. #24, 300223 Timisoara, Romania; floricabalanean@yahoo.com; 4 Research Center for Complex Fluids Systems Engineering, Politehnica University of Timisoara, M. Viteazu Ave. #1, 300222 Timisoara, Romania

**Keywords:** magnetic paper, magnetic white paper, superparamagnetic paper, microfiber, cellulose, electrospinning, iron oxide magnetic nanoparticles

## Abstract

The preparation procedure of zero magnetic remanence superparamagnetic white paper by means of three-layer membrane configuration (sandwiched structure) is presented. The cellulose acetate fibrous membranes were prepared by electrospinning. The middle membrane layer was magnetically loaded by impregnation with an aqueous ferrofluid of 8 nm magnetic iron oxide nanoparticles colloidally stabilized with a double layer of oleic acid. The nanoparticles show zero magnetic remanence due to their very small diameters and their soft magnetic properties. Changing the ferrofluid magnetic nanoparticle volume fraction, white papers with zero magnetic remanence and tunable saturation magnetization in the range of 0.5–3.5 emu/g were prepared. The dark coloring of the paper owing to the presence of the black magnetite nanoparticles was concealed by the external layers of pristine white cellulose acetate electrospun fibrous membranes.

## 1. Introduction

Magnetic paper has constantly attracted considerable attention due to its potential application in a wide range of technologies including information storage [1], electromagnetic shielding, magnetographic printing, magnetic filtering, and security paper [2]. Different preparation methods have been developed towards the generation of magnetic paper using magnetic nanoparticle-based fillers: Direct wet end addition, lumen loading, in situ magnetic particle synthesis, and fiber nanocoating [3]. For this purpose, a wide range of magnetic fillers were considered: Bare nanoparticles [2,4], organic capped nanoparticles [5], single core-(oxide)shell particles [6], multicore-(oxide)shell particles [7], and fibers [8].

Due to the inherent high light absorbance of ferromagnetic and ferrimagnetic materials, magnetic loading leads to paper coloring. The coloring becomes more pronounced upon increasing the magnetic loading. Attempts have been made to alleviate this problem by tailoring the optical properties of the magnetic fillers [5,7,9], but no satisfactory compromise between magnetic loading and coloring degree was achieved so far. A technologically feasible solution to avoid coloring is sheet sandwiching, which is widely used in the paper and cardboard industry. This solution was recently explored by Sriplai and co-workers [8], who significantly reduced the coloring by sandwiching a 20 μm bacterial cellulose sheet impregnated with a ferromagnetic material between two layers of pristine 20 µm bacterial cellulose white sheets. Thus, the white magnetic paper itself is a ~60 µm three-layer cellulose composite with standard whiteness and a high degree of magnetic loading (~20 emu/g saturation magnetization). A drawback due to the use of cobalt ferrite nanoparticles is the rather high ~5 emu/g remanent magnetization and ~200 Oe coercive field, which is an important limiting factor in certain applications like electromagnetic shielding, magnetic filtering, or security paper.

In the present study, we aimed to prepare white magnetic paper with zero magnetic remanence. This is achieved by means of electrospun cellulose microfibers doped with superparamagnetic iron oxide nanoparticles for the preparation of the magnetically loaded mid layer of the white paper sandwich composite. Electrospinning is considered to be one of the most versatile fabrication methods enabling the production of functional nano- and microfibers of various chemical compositions, controlled orientations, and morphologies [10,11]. The electrospinning technique is simple, cost-effective, and industrially scalable [11,12], providing a straightforward way to produce long and continuous polymer fibers by using electrical forces. Moreover, electrospinning enables the incorporation of a variety of inorganic nanoparticles within or onto the fibers’ surfaces [13], thus generating electrospun fibrous nanocomposites [14,15,16,17,18,19,20]. A distinct advantage of the fabrication methodology employed in the present study, making use of cellulose acetate electrospun fibers as host matrices for the anchoring of magnetic nanoparticles instead of conventional filter (cellulose) paper, is that the former are characterized by higher surface area and higher porosity due to their open pore structure, thus allowing the anchoring of increased amounts of MIONPs [21]. As a consequence, materials exhibiting higher magnetization can be obtained. In addition, unlike the case of cellulose-based filter papers consisting of highly crystalline cellulose [22], the lower degree of crystallinity of cellulose acetate (CA) [23] further facilitates the post-magnetization process.

The produced magnetoactive white paper was characterized by means of FTIR, scanning electron microscopy, and magnetometry, while the latter verified the superparamagnetic behavior of the produced paper exhibiting tunable magnetization upon varying the magnetic content and zero magnetic remanence.

## 2. Materials and Methods

### 2.1. Chemicals and Reagents

Cellulose acetate (CA) (Μn _~_ 30,000, *Sigma-Aldrich,* St. Louis, Missouri, USA, no purity info), acetone (extra pure, ≥99.5%, Scharlau, Barcelona, Spain), sodium hydroxide (NaOH, M = 40, ≥99%, Scharlau, Barcelona, Spain), and ethanol (KGaA ≥99.9%, Merck, Kenilworth, New Jersey, USA ) were used as received from the manufacturer. Iron (II) and Iron (III) chloride (, Merck, Kenilworth, New Jersey, USA, no purity info), NaOH (, Merck, Kenilworth, New Jersey, USA, no purity info) were used for the synthesis of the magnetic iron oxide nanoparticles (**MIONP**s). Oleic Acid (OA) (90%, *Sigma-Aldrich*, St. Louis, Missouri, USA) was used for the hydrophilic colloidal stabilization of the nanoparticles (**MIONP-OAOA**).

### 2.2. Synthesis of Water-Based Ferrofluid

Water-based ferrofluid containing MIONPs was prepared (30 mL). The magnetite nanoparticles, obtained by chemical coprecipitation, were colloidally stabilized with a hydrophilic double layer of oleic acid (OA) molecules [24]. The MIONP volume fraction in the ferrofluid was 2.5%.

### 2.3. Fabrication of Electrospun CA Fibrous Membranes

Initially, a solution of CA (solution concentration: 12.5% *w*/*v*) was prepared in acetone and it was left to stir overnight to ensure complete dissolution of the polymer.

**Figure 1:**
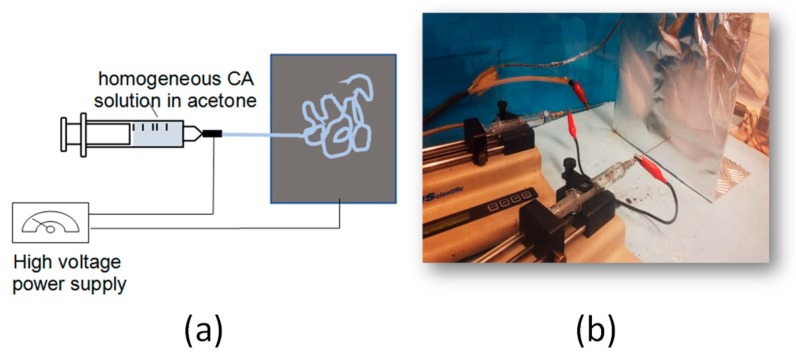
Schematic representation (**a**) and photograph (**b**) of the electrospinning set-up. The latter shows two spinnerets connected in series. Upon applying a high voltage, cellulose acetate (CA) fibers are ejected from the spinnerets and they are deposited onto the metallic collector. Electrospinning conditions: CA solution in acetone: Solution concentration 12.5% *w*/*v*; needle diameter: 16 G; applied voltage: 15 kV; needle-to-collector distance: 10 cm; solution flow rate: 5.9 mL/h.

Fibrous CA membranes were prepared by electrospinning. The electrospinning set-up employed was a conventional custom-made system that included the following equipment: A high-voltage power generator (ES50P-20W, Gamma High Voltage Research, Ormond Beach, Florida, USA); an automatic syringe/flow pump (KDS 789252, KD Scientific Inc., Holliston, Massachusetts, USA); a glass syringe of 10 mL connected with metal spinneret needle; a flat metal collector (282 mm length × 279 mm height); Ground and Power supply electrodes; and an enclosure safety interlocked Faraday Plexiglass chamber (Figure 1).

CA (1.25 g) was dissolved in acetone (10 mL) by magnetic stirring (IKA magnetic stirrer—RCT basic, rotation speed: 300 rpm) at room temperature. The CA/acetone homogeneous solution was then loaded into a 10 mL glass syringe equipped with a spinneret needle (16 G). The syringe was placed on the flow pump with specially connected spinneret needle electrode for positively charge (several kV potential) supply. The solution was then dispensed via the automatic syringe/flow pump by setting the ideal electrospinning conditions and electrospun CA fibers were collected onto the grounded metallic collector. Electrospinning experiments were performed at room temperature (25 ± 2 °C) and relative humidity 50 ± 5% *RH*. The optimum electrospinning conditions to obtain uniform bead-free fibers were the following: Applied voltage: 15 kV; flow rate: 5.9 mL/h; needle-to-collector-distance: 10 cm.

### 2.4. Fabrication of Magnetic, Cellulose-Based White Paper in the Form of a Sandwiched Structure

**Figure 2:**
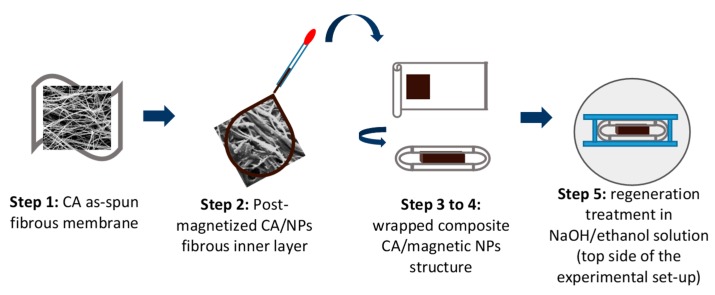
Schematic representation of the preparation steps followed for generating the composite CA/MIONP-OAOA _CA/CA sandwiched structure.

**Figure 3:**
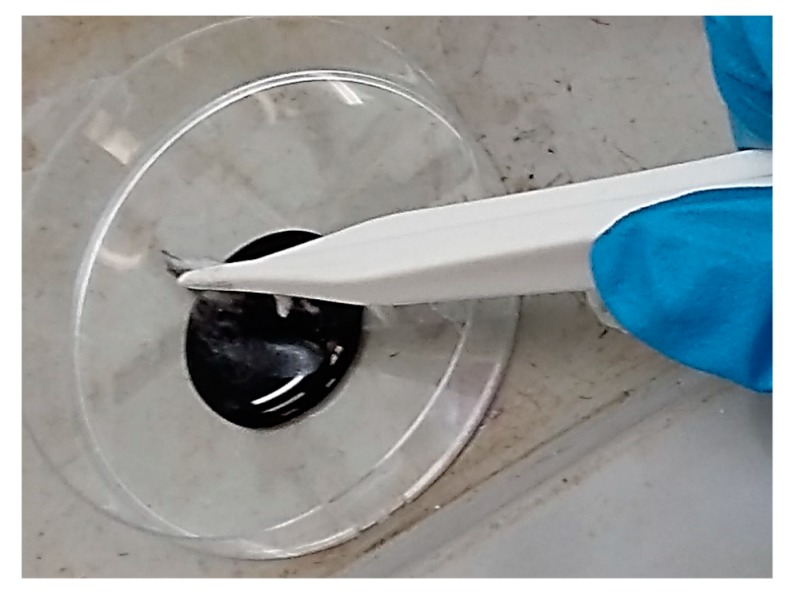
Photograph of the post-magnetization step involved in the preparation of the inner magnetic layer of the CA/MIONP-OAOA _CA/CA sandwiched structure.

The procedure followed for the fabrication of superparamagnetic white paper having a sandwiched structure consisted of five steps and it is described in the following:

**Step 1:**  CA electrospun fibrous membrane was fabricated by electrospinning under the optimum electrospinning conditions. The duration of the electrospinning process was 1 h. Then, the as-prepared CA membrane was cut in small rectangular layers (0.1 mm × 0.1 mm, inner layers of the sandwiched structure) followed by a post-magnetization procedure.
**Step 2:**  The post-magnetization procedure involved the impregnation of the CA fibrous mat with a specific amount of an aqueous solution of MIONP-OAOA NPs via drop casting. More precisely, four magnetic aqueous solutions were prepared (**A**: As-synthesized mother solution; **B**: 2× dilution of the mother solution; **C**: 5× dilution of the mother solution; **D**: 10× dilution of the mother solution). Each diluted aqueous magnetic amount (i.e., 2×; 5×; 10×) was rapidly stirred by vortex for ensuring homogeneity.
**Step 3:**  CA electrospun fibrous membrane was fabricated by electrospinning (process duration: 2 h). The as-prepared CA membrane was cut in orthogonal layers (length × width: 1.2 mm × 0.25 mm, outer layer of the sandwiched structure, mass of outer layer = 100 mg). Eight samples were prepared in total.
**Step 4:**  The generation of the sandwiched structure was realized by wrapping the magnetic inner layer (produced as described in step 2) within the CA outer layer produced in step 3. The final sandwiched structure was initially fixed with glassy rods that were placed at the 2 edges of the specimen, followed by the placement of the latter in between two glass slides as schematically depicted in Figure 2. The number of the outer layers may vary, thus controlling the overall thickness of the final composite.
**Step 5:**  The final step involved the regeneration of CA into cellulose by the immersion of the generated sandwiched structure in NaOH/ethanol solution (0.1 M, 60 mL solution) for 48 h (under airtight closure) as presented in Figure 3.

Four magnetic paper samples were prepared using the magnetically loaded CA fibrous layers described in **Step 2** above and Table 1: S0×, S2×, S5×, and S10×.

### 2.5. Characterization

#### 2.5.1. Transmission Electron Microscopy (TEM)

TEM was performed on a Hitachi HD-2700 scanning transmission electron microscope (STEM), equipped with a cold field emission gun, working at an acceleration voltage of 200 kV. Images were obtained using the Digital Micrograph software from Gatan. A drop of 5 µL ferrofluid was deposited on a copper grid coated by a thin carbon film and left to dry prior to TEM analysis.

#### 2.5.2. X-Ray Diffraction (XRD)

X-Ray diffraction (XRD) measurements were performed with a D8 Advance X-Ray diffractometer, Bruker, Billerica, Massachusetts, USA, with a Ge (111) monochromator for Cu-*K*α1 radiation (*λ* = 1.5406 Å) with the source power of 40 kV and 40 mA, at room temperature, and LynxEye position sensitive detector.

#### 2.5.3. Scanning Electron Microscopy (SEM)

The morphological characteristics of the as-prepared CA electrospun fibrous layer, the magnetically modified CA inner fibrous layers, and the final sandwiched composite structures (prior to and after regeneration) were investigated by Scanning Electron Microscopy (SEM) (Vega TS5136LS-Tescan, Brno, Czech Republic) using a Secondary Electron detector (SE detector) at 20 (kV) High Voltage (HV). The samples were placed on small circular SEM sample holders and they were sputter-coated with a thin gold layer using a sputtering system (K575X Turbo Sputter Coater-Emitech, Quorum Technologies Ltd., West-Sussex, UK) at 40 (mA) prior to visualization. The average fiber diameters were measured using an image analysis software (ImageJ ver.1.42q, National Institutes of Health, Bethesda, Maryland USA by measuring at least 25 fibers from the SEM images.

#### 2.5.4. Fourier-Transform Infrared (FTIR) Spectroscopy

Fourier-Transform Infrared (FTIR) Spectroscopy was performed with FTIR-NIR Prestige-21 spectrometer system, Shimadzu Corporation, Kyoto, Japan in a wavenumber range of 4000–700 cm^−1^ to investigate the success of the CA regeneration process. After the CA-based fibrous mats (dimensions: 0.1 mm length × 0.1 mm width) were immersed in NaOH/ethanol solution, they were repeatedly washed with de-ionized water, and upon drying, they were analyzed under ambient conditions.

#### 2.5.5. Magnetometry

The static magnetization of the samples was measured by means of vibrating sample magnetometry on a *VSM880* magnetometer, *ADE Technologies inc., Lowell, MA USA* at room temperature in ±1000 kA/m magnetic field range. For the purpose of magnetization measurements, 3 mm disks of magnetic paper from the samples were obtained using a paper hole puncher.

## 3. Results and Discussion

### 3.1. Magnetic Nanoparticle Characterization

**Figure 4:**
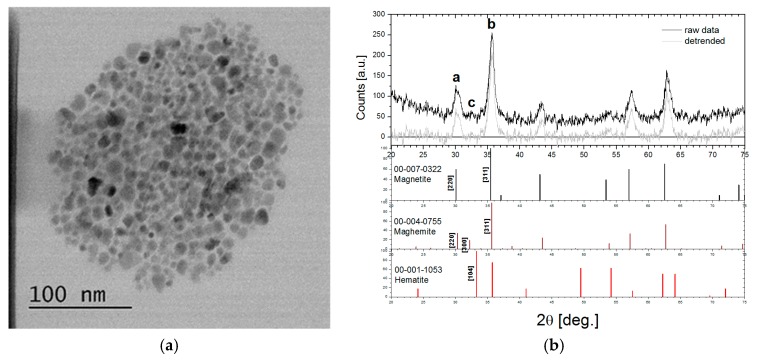
(**a**) TEM image of the MIONPs, (**b**) XRD spectrum of the MIONPs and magnetite, maghemite, and hematite PDFs.

The TEM image of the magnetic nanoparticles from the structure of the water-based ferrofluid are presented in Figure 4a. The diameter of the magnetic nanoparticles was measured on a sample of 200 nanoparticles using ImageJ [25]: 8.2 ± 1.9 nm.

The XRD spectrum of the surfactant-coated magnetic nanoparticles is shown in Figure 4b. The absence of [104]-Hematite peak excludes the presence of hematite in our sample. The ratio of peaks **a** and **b** amplitude (~1/3) is closer to maghemite (1/3) than magnetite (6/10), but [300]-Maghemite is insufficiently expressed in peak **c**. Therefore, we conclude that the MIONPs are a mixture of magnetite and maghemite.

**Figure 5:**
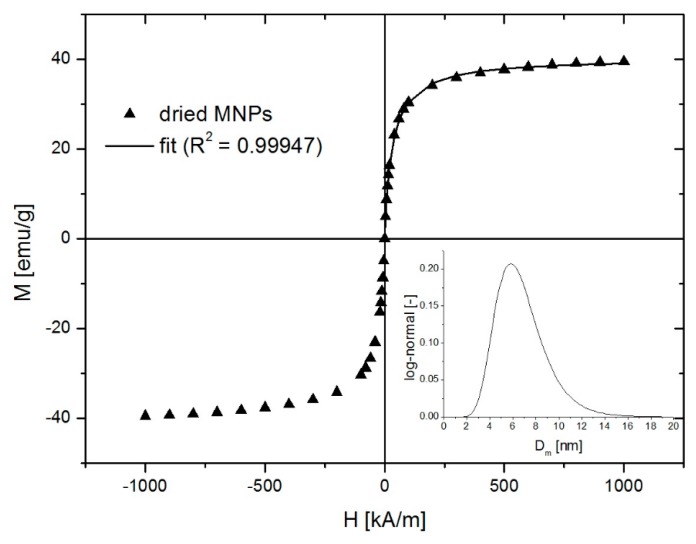
Magnetization plot of dried magnetite nanoparticles.

In the frame of our research, the most important characteristic of the MIONPs is their magnetic properties. Figure 5 presents the magnetic field dependence of the dried MIONPs from the water-based ferrofluid. The saturation magnetization of the dried MIONP sample was lower than that of bulk magnetite, first because of the surfactant and second due to the non-magnetic layer at the surface of the nanoparticles. The magnetic diameter distribution of the nanoparticles was obtained by magnetogranulometry, by means of nonlinear regression of the magnetization curve with the second-order modified mean field theory developed by Ivanov and co-workers for highly concentrated polydisperse samples [26,27]. The nanoparticles’ magnetic diameter distribution was assumed log-normal [28]:

(1) $$f\left(D\right)=\frac{1}{D\sigma \sqrt{2\pi }}\cdot e^{-\frac{1}{2\sigma^{2}}\cdot {\left(\ln\frac{D}{D_{0}}\right)}^{2}}$$

where *D*_0_ and *σ* are the fit parameters. Using *D*_0_ and *σ*, the calculation of MNP’s mean magnetic diameter (<D>) and standard deviation (δD) is straight forward. The fitting curve with an excellent R^2^ of 0.99947 is presented in Figure 5 (solid line) and the resulting magnetic diameter log-normal distribution with *D*_0_ = 6.43 nm and *σ* = 0.31 obtained from the fit is presented in the inset. The calculated magnetic diameter was 6.8 ± 2.2 nm. Such a small value of the mean magnetic diameter of the magnetite nanoparticles explains the absence of the magnetic remanence even in the highly packed dry state. The magnetic diameter was smaller than the physical diameter determined from TEM due to the nonmagnetic layer at the surface of the nanoparticles.

### 3.2. Fabrication of Electrospun CA Fibrous Mats

**Figure 6:**
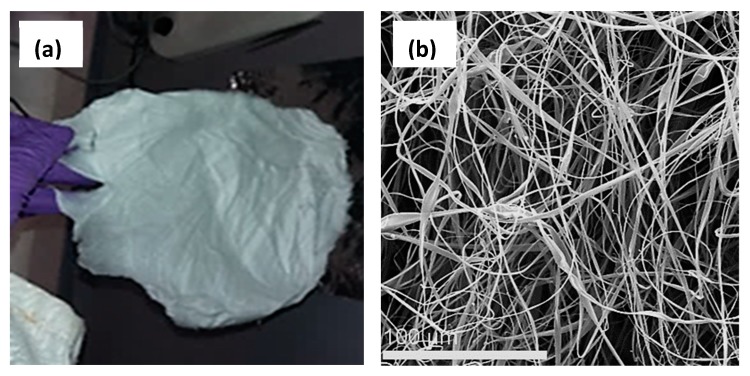
(**a**) Photograph of the CA fibrous mat produced by electrospinning. Electrospinning conditions: Applied voltage: 15 kV; flow rate: 5.9 mL/h; needle-to-collector-distance: 10 cm. (**b**) Representative SEM image of the as-prepared CA fibers (SEM operation conditions: HV: 20 KV; MAG: 800×; Working distance: 9.4521 mm).

Homogeneous, electrospun CA fibrous mats were initially prepared by following the optimum electrospinning conditions as described in the experimental section (Section 2.3). Figure 6a provides a photograph of the as-prepared CA fibrous mat. Electrospinning was conducted for 1 h resulting to a mat thickness of 0.350 mm. The latter may vary, upon altering the electrospinning time.

The morphology of the as-prepared CA fibers was visualized by SEM and representative images are provided in Figure 6b. The average diameters of the produced CA fibers were calculated to be 2.942 ± 0.842 μm.

### 3.3. Post-Magnetization Process

**Figure 7:**
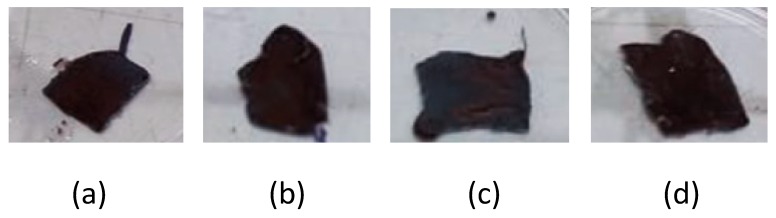
Photographs of magnetically modified electrospun CA fibrous mats. The modification step was realized via drop casting of a specific amount of MIONP-OAOA aqueous solutions onto the fibrous mats. The four samples (**a**–**d**) were prepared by drop casting of magnetic aqueous solutions of variable concentrations as follows: Sample a—drop casting of the as-synthesized mother solution; sample b—drop casting of 2× dilution of the mother solution; sample c—drop casting of 5× dilution of the mother solution; sample d—drop casting of 10× dilution of the mother solution.

The post-magnetization of the inner CA electrospun fibrous layers was accomplished by following a simple modification step involving the impregnation of the CA fibrous mat with a specific amount of aqueous solution of preformed MIONP-OAOA NPs that were synthesized as described in Section 2.2. Upon impregnation, the color of the CA fibers immediately changed from white to dark brown as seen in Figure 7.

**Figure 8:**
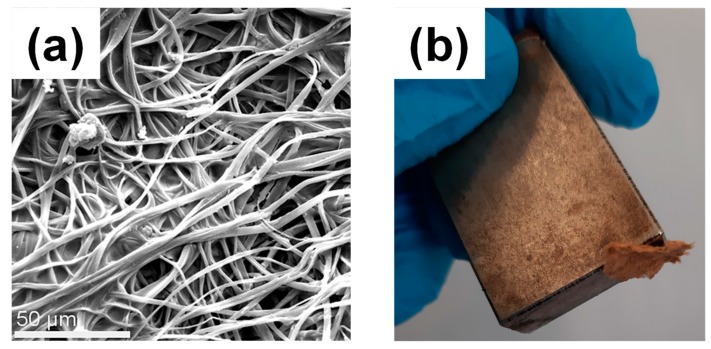
(**a**) Representative SEM image of the magnetically modified CA fibers decorated with *MIONP-OAOA* NPs. (**b**) Photograph of the magnetically modified membrane attracted by a magnet.

The morphological characteristics of the magnetically modified CA fibers were investigated by SEM. The average fiber diameters were found to be 3 ± 1 μm, which is very similar to that of the pristine CA fibers. Moreover, the magnetic character of the magnetically modified CA fibrous mats was confirmed by the fact that the resulting post-magnetized fibrous mats were attracted by an external magnet (Figure 8b).

### 3.4. Fabrication of Sandwiched CA/MIONP-OAOA _CA/CA Composites and Cellulose-Based Regenerated Analogues (Cellulose-Based White Magnetic Paper)

**Figure 9:**
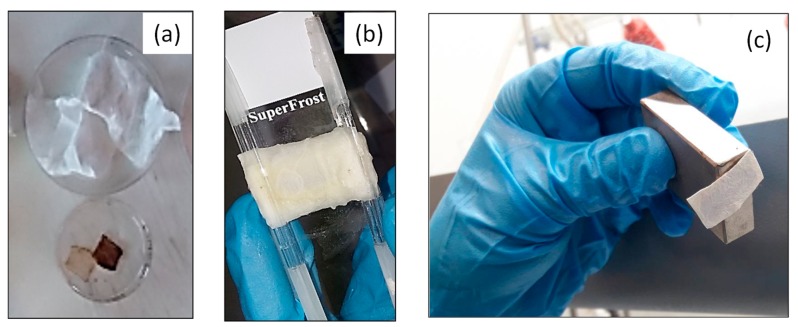
Consecutive preparation steps followed for the generation of cellulose-based white-colored magnetic paper with a sandwiched structure. As-prepared CA electrospun membrane employed as the outer layer and inner MIONP-OAOA _CA magnetic layer (**a**). Wrapping of the magnetic inner layer within the CA outer layer and fixation of the sandwiched structure with glassy rods followed by their placement in between two glass slides (**b**). Magnetic white paper attracted by a permanent magnet (**c**).

The nanocomposite magnetically modified CA fibrous mat (inner layer) was wrapped within a second CA fibrous mat of higher thickness (outer layer) as described in the experimental section (Section 2.4). The produced nanocomposite sandwiched structure was further treated with NaOH/ethanol solution to obtain the final cellulose-based regenerated analogues. Figure 9 provides photographs of the consecutive preparation steps followed as described in Section 2.4, resulting in the generation of cellulose-based, white-colored magnetic paper.

**Figure 10:**
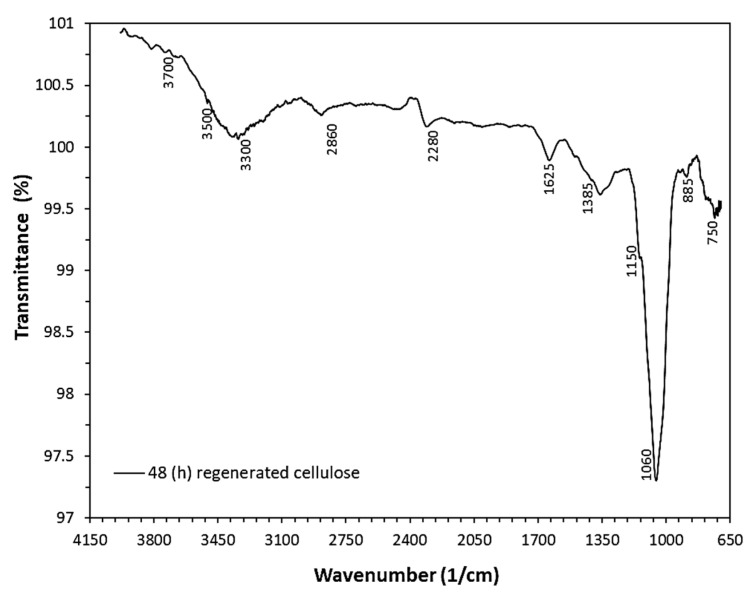
FTIR spectrum of regenerated cellulose fibers obtained upon immersion of CA fibers in NaOH/ethanol solution for 48 h.

The success of the regeneration process was verified by FTIR. As seen in Figure 10, the presence of characteristic FTIR bands appearing at 3300–3700 cm^−1^ corresponded to the stretching vibrations of O-H group, which can be directly correlated to the chemical structure of pure cellulose. Moreover, the absence of the characteristic absorption bands in the FTIR spectrum of the regenerated material, appearing in the case of CA at 1770 cm^−1^ (C=O), 1385 cm^−1^ (CH_3_) and (900–1300 cm^−1^) (C-O), further verifies the success of the regeneration treatment [29].

**Figure 11:**
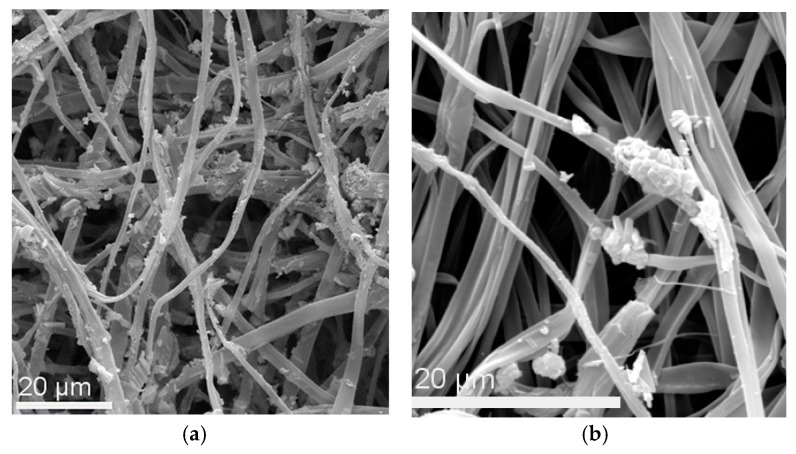
(**a**) SEM images of the magnetically modified regenerated cellulose fibers decorated with MIONP-OAOA NPs (inner layer). (**b**) Magnetically modified, regenerated cellulose fibers (nanocomposite sandwiched structure).

SEM was employed to study the morphology of the regenerated nanocomposite cellulose_ MIONP-OAOA inner layer and of the final regenerated nanocomposite sandwiched structure (Figure 11a,b, respectively). As seen in Figure 11a, the presence of the MIONP-OAOA NPs was clearly observed onto the fibers’ surfaces corresponding to the inner cellulose-base magnetic layer, whereas their presence became less obvious in the sandwiched nanocomposite analogue as expected, owing to the presence of the outer cellulose fibrous layer.

**Figure 12:**
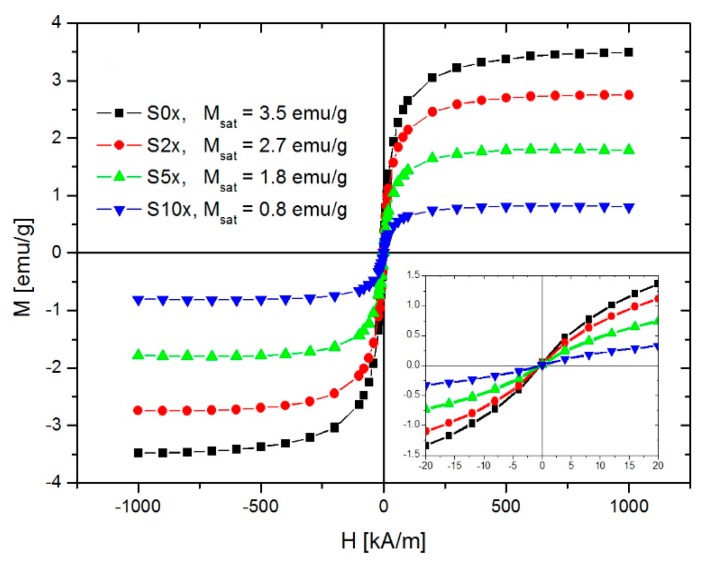
Magnetization plots of the magnetic papers having variable magnetic loadings.

Figure 12 presents the magnetic field dependence of the samples’ mass magnetization. All samples showed superparamagnetic behavior and none of the samples showed magnetic remanence. As shown above, this is due to the magnetic properties of the MNPs used in the preparation of the magnetic mid-layer. Furthermore, as seen from the figure, the saturation magnetization decreased with increasing dilution of the ferrofluids used to magnetically load the CA-fibers. The saturation magnetization did not change linearly with the dilution ratio mainly due to the presence of the outer layer non-magnetic mass and also because the nanoparticle retention in the CA-fibers was not linearly correlated with the ferrofluid volume fraction, as can be seen in Table 1. The saturation magnetization of the magnetic paper can be increased by either improving the MNPs retention and/or increasing the thickness of the magnetic mid-layer.

## 4. Conclusions

Zero magnetic remanence and white coloring of magnetic paper can be achieved by incorporating magnetite nanoparticles with diameter below 10 nm within electrospun cellulose fibrous membranes in a sandwiched, three-layer configuration. Cellulose fibrous membranes serve both as a host for magnetite nanoparticles and a coloring concealment. Fabrication of superparamagnetic white paper with saturation magnetization values can be obtained upon altering the magnetic content of the middle layer. Future work involved the development of alternative routes facilitating a better control over the paper’s magnetic loading.

## Figures and Tables

**Table 1:** Details of materials’ quantities used in the preparation of the inner magnetic CA fibrous layers for different magnetic iron oxide nanoparticles (MIONP)-OA. Oleic Acid (OA) solution concentrations. (2×, 5×, and 10× denotes the times of dilution of the mother 0× solution with deionized water). Mother solution 0× concentration: 1.0703 g/cm^3^.

Quantities\Dilution	0× (Mother Solution)	2×	5×	10×
dry CA mass (mg)	3.8	3.4	3	2.7
NP solution (μl)	57	51	45	41
final dry magnetic CA mass (mg)	8.6	7	4.3	3
Magnetic loading (mg)	4.8	3.6	1.3	0.3
Magnetic loading (%)	55.8	51.4	30.2	10
